# Benthic Fluxes of Fluorescent Dissolved Organic Material, Salt, and Heat Measured by Multiple-Sensor Aquatic Eddy Covariance

**DOI:** 10.3390/s22228984

**Published:** 2022-11-20

**Authors:** Irene H. Hu, Harold F. Hemond

**Affiliations:** 1 Department of Civil and Environmental Engineering, Massachusetts Institute of Technology, Cambridge, MA 02139, USA; 2 Monterey Bay Aquarium Research Institute, Moss Landing, CA 95039, USA

**Keywords:** aquatic eddy covariance, benthic flux, fluorescent dissolved organic material, submarine groundwater discharge

## Abstract

Aquatic eddy covariance (AEC) is an in situ technique for measuring fluxes in marine and freshwater systems that is based on the covariance of velocity and concentration measurements. To date, AEC has mainly been applied to the measurement of benthic oxygen fluxes. Here, development of a fast multiple-channel sensor enables the use of AEC for measurement of benthic fluxes of fluorescent material, salt, and heat at three distinct sites in Massachusetts, USA, including the Connecticut River, the Concord River, and Upper Mystic Lake. Benthic fluxes of salt, useful as a tracer for groundwater input (submarine groundwater discharge), were consistent with independent measurements made with seepage meters. Eddy fluxes of heat were consistent with the balance of incoming solar radiation and thermal conduction at the sediment surface. Benthic eddy fluxes of fluorescent dissolved organic material (FDOM) revealed a substantial net downward flux in the humic-rich Concord River, suggesting that microbial consumption of dissolved organic carbon in the sediment was significant. Simultaneous measurement of several fluxes expands the utility of AEC as a biogeochemical tool while enabling checks for mutual consistency among data channels.

## 1. Introduction

Knowledge of benthic fluxes is important for understanding both natural biogeochemical cycles as well as the fate and transport of contaminants in marine and freshwater ecosystems. However, benthic fluxes are typically difficult to measure. Aquatic eddy covariance (AEC), based on the covariance of rapid and collocated velocity and concentration measurements [1], has demonstrated great value as a minimally invasive, in situ technique for benthic flux measurement. Major advances have been made in its development and application, especially with respect to the measurement of oxygen fluxes [2]. Other fluxes have also been measured using AEC, including electrical conductivity [3], bisulfide [4], nitrate [5], and pH [6,7]. Here, we present field trials of an eddy covariance instrument using the multi-function sensor FACT (Fluorescence and Conductivity, Temperature), previously described by Hu and Hemond [8]. The three fluxes measured by the instrument are thus of fluorescent material, ionic material contributing to electrical conductivity (dissolved salts), and heat.

Fluorescent material whose benthic fluxes are of interest include a variety of pollutants, such as certain polycyclic aromatic hydrocarbons (PAHs), which can be released into surface waters from contaminated sediments, as well as naturally occurring fluorescent dissolved organic material (FDOM). Here, the wavelengths of the FACT sensor were chosen to target FDOM. FDOM measurements can be extrapolated to concentrations of dissolved organic material (DOM) or dissolved organic carbon (DOC) via site-specific and seasonal relationships [9,10,11]. Thus, measurement of benthic fluxes of FDOM can provide insight into the biogeochemical roles of DOC and related dynamics [12,13], as well as carbon budgets and carbon cycling in aquatic and marine systems [14,15]. 

Benthic fluxes of electrical conductivity can be used to infer benthic fluxes of dissolved salts which, in cases where sediment porewaters have a different salinity than overlying water, can provide a direct estimate of groundwater input to surface water [3]. The relationship between total salt concentration and electrical conductivity in water is influenced by both temperature and salt composition, both of which may need to be accounted for in certain situations; however, as a tracer substance, total salt concentration is nearly conservative. Thus measuring the benthic flux of salt can enable estimates of groundwater and, by extension, fluxes of other biogeochemicals whose movement is dominated by advective transport via groundwater [16]. 

Benthic fluxes of heat can also be used as a groundwater tracer, similar to salinity [3], or can help inform an understanding of the hydrodynamics of a surface water body. They have previously been measured alongside other fluxes using AEC [17], yielding insights about benthic heat dynamics [18], and in some cases used to correct signals of the concentration sensor [19]. In the present case, heat flux provides a cross-check on the measurement of other benthic fluxes, i.e., comparing a benthic eddy heat flux against other heat flux measurements may help identify errors or improve confidence in the system’s performance.

The FACT sensor operates with an analyte sensing volume for conductivity and fluorescence that is displaced from the physical structure of the probe tip, and thus can be made to overlap with the velocity sensing volume without interfering with velocity measurements. This closer overlap between sensing volumes avoids the need for time shift corrections due to sensing volume displacement, as well as uncertainties that increase as the horizontal velocity direction departs from a downstream sensor orientation [20,21]. 

The field deployments described here are intended to test and demonstrate the performance of a benthic flux AEC system incorporating the multi-function FACT sensor in several river and lake environments. While a key objective is to demonstrate utility of a fast fluorescence sensor in AEC applications, the deployments also serve to determine the ability of the previously described FACT sensor to function under the rigors of field conditions, and to examine the degree of synergism gained through the intercomparison of multiple channels of data. In addition, certain site-specific biogeochemical observations are documented. 

## 2. Materials and Methods

### 2.1. Instrumentation

**Figure 1:**
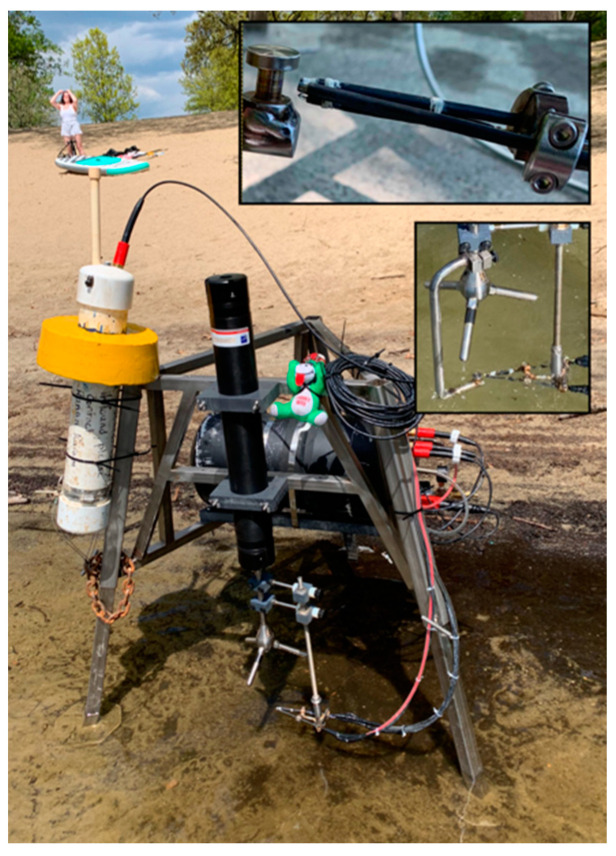
Eddy flux instrument mounted on benthic lander. Communication buoy, shown strapped to the lander for very shallow deployments, floats free for deeper deployments. (**Top inset**) FACT sensor head including optical fibers, conductivity electrodes, and thermistor. (**Bottom inset**) FACT sensing head shown with jig for aligning sensing volumes.

The EC flux instrument comprises a Nortek Vector acoustic Doppler velocimeter (ADV) paired with a novel three-channel sensor measuring fluorescence, electrical conductivity, and temperature in nearly the same sensing volume (Figure 1). This multi-function sensor, abbreviated FACT (Fluorescence and Conductivity, Temperature), is described in detail by Hu and Hemond [8]. 

Briefly, the fluorescence sensor is an optical fiber spectrofluorometer capable of high-speed, high-resolution, in situ measurements. The instrument excites fluorescence in a sensing volume that is projected from optical fibers and hence can overlap closely with the ADV sensing volume, despite the probes being located at an offset. The sensitivity of the instrument was improved relative to that reported earlier by replacing the 375 nm light-emitting diode (LED) with a high-power model of the same emission wavelength (Thorlabs M375L4) paired with a custom LED driver and a pair of aspheric condenser lenses (Thorlabs ACLU2520U-A) for focusing diverging LED light into a 1000 um silica optical fiber. A second fiber transmits fluorescence light from the sensing volume to a tunable monochromator (Spectral Products CM110), from which light enters a fast photomultiplier tube (ET Enterprises 911WB). Updated custom circuitry enables photon counts of over 30 million photons/s, thus providing high sensitivity and dynamic range as well as improving the ability of the instrument to reject ambient light using its modulation feature.

The temperature sensing channel uses a fast-responding miniature NTC thermistor (Amphenol FP07DB154N) packaged with epoxy resin in stainless steel tubing and encased by electrical coating (3M Scotchkote^TM^) and dual-wall heat shrink tubing, but with the sensing tip exposed to water. The thermistor probe is fixed to the sensing end of one of the optical fibers. 

The conductivity cell is formed directly by the tips of the 15 cm stainless steel tubings protecting the sensing ends of the optical fibers. Both tubings (each housing a fiber) are exposed for a length of approximately 1.5 mm at the tip, with their inside-facing edges coated with insulating epoxy. The remainder of each tubing is encased in Scotchkote^TM^ and dual-wall heat shrink tubing. 

The open-path spectrofluorometer cell, open-path conductivity cell, and thermistor are integrated into a single sensor head which is positioned such that a large portion of the fluorescence cell and a significant portion of the conductivity cell lie within the sensing volume of the ADV, while the thermistor is positioned on the periphery (Figure 1).

The microcomputer controlling all FACT concentration measurements is used to communicate with the ADV and coordinate measurements using the ADV’s TTL ‘Synch In’ signal. Thus, velocity and concentration measurements are fully integrated for eddy covariance measurements, which are initiated by the user via remote login. Once deployed, the instrument communicates with shore via an RS-232 radio buoy similar to that described by Gardner et al. [22]. 

For field experiments, the instrument was mounted on a 3-legged benthic lander (Figure 1) fabricated of square stainless steel tubing, with adjustable legs and 7.5 cm hexagonal foot pads. The FACT sensor head was adjusted such that the probe tips were 1–2 mm outside of the ADV sensing volume, with interference confirmed to be adequately low via examination of ADV data [8,17]. The measurement height was positioned 7–16 cm above the sediment floor. Relatively low measuring heights were chosen for these deployments to maximize concentration gradients [23] and thus lower the sensitivity requirements of the concentration sensors. Measuring closer to the bottom would reduce the effect of storage and horizontal transients, recognizing tradeoffs with the ability to average over heterogeneity [24,25]. Optical fibers and cables were zip tied to the lander body to avoid motion in the presence of significant current or wave action, and the setup was deployed via wading.

**Figure 2:**
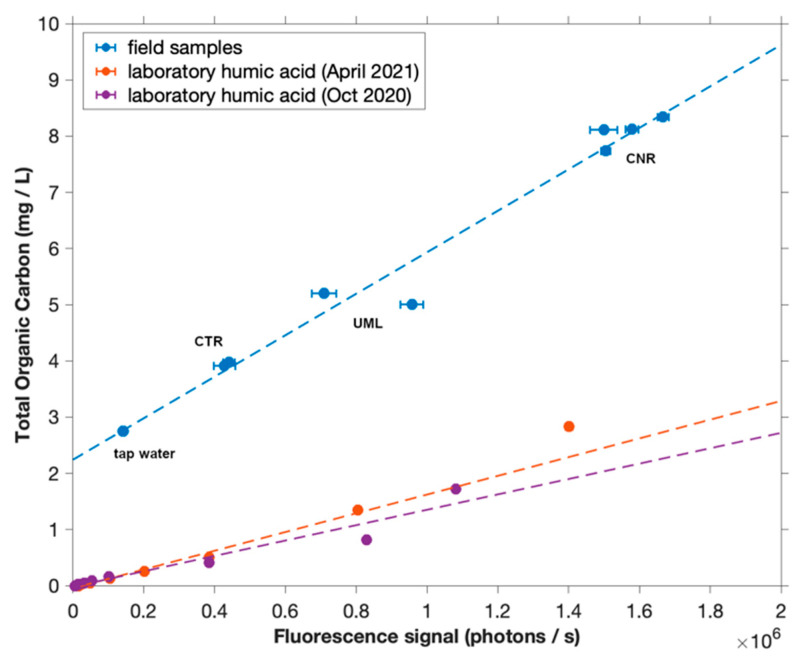
Calibration of photon counter using in situ field measurements compared to TOC of grab samples as measured by analyzer. Calibrations with laboratory Sigma Aldrich humic acid are shown for comparison.

Spectral scans were obtained at each site, before setting the monochromator to 465 nm for eddy covariance measurements. The LED was modulated at 1000 Hz to reject ambient light while minimizing artifacts from failing to capture rapid changes in fluorescence. Field observations of fluorescence are reported as mg/L DOC using a linear calibration curve determined using surface water samples from all three field sites. Samples from field sites were found to have consistent fluorescence to total organic carbon (TOC) ratios (which nevertheless differed substantially from Sigma Aldrich laboratory humic acid) (Figure 2). TOC is considered equal to DOC, as no difference in measured TOC concentration was observed after filtering through combusted glass fiber filters. Fluorescence is thus calibrated to DOC and the resulting benthic fluxes of fluorescent materials as measured by AEC are presented as fluxes of DOC; the validity of this assumption is discussed further in Section 4.3.2.

Temperature measurements and conductivity measurements were calibrated in laboratory and found to be consistent with field measurements. Conductivity measurements were compensated to 25 °C, using temperature compensation coefficients of variation estimated from laboratory measurements of mixtures of tap water and ice heated in small increments. These coefficients, which can vary with temperature and solute [26], were calculated for different temperature ranges (spanning 5 °C each) and applied to each field trial accordingly.

### 2.2. Data Analysis

For eddy covariance, measurements were made at 48 Hz and averaged to 16 Hz. Prior to averaging, velocity data were screened, in most cases for correlation >70% and SNR > 5, except for the Connecticut River site where a correlation threshold of 60% was used (see Section 3.3). Velocity data were despiked using the acceleration method [27], with thresholds of g for x and y orientations and 0.3 g for the vertical z direction (where g = 9.8 m/s^2^). For all deployments, the despiking algorithm did not identify any spikes in velocity data, due in part to a conservative choice of velocity range during deployment [28]. However, spikes in fluorescence and velocity data at the Concord River site, and in conductivity data at the Connecticut River site, were manually identified and removed (see Section 3.3). After averaging, missing data were replaced by values linearly interpolated from the closest points [27]. 

Velocity data were not rotated even on sloped surfaces, as all lander legs were set to the same height and the lander is designed to hold the ADV perpendicular to the floor. Standard coordinate rotation algorithms, such as double rotation and planar fit [29,30], produced unrealistic angles, possibly due to the lower velocities observed at these sites. Thus, for these deployments, not rotating the data was deemed the best method to align the xy plane of the coordinate system approximately parallel to the bottom surface.

A time shift correction was not applied, despite the small (1–2 mm) displacement of the thermistor head from the ADV sensing volume, as the displacement was typically perpendicular to flow and a travel time alignment is therefore not applicable. However, the displacement is small enough that decoupling between velocity and temperature measurements was likely small [20]. 

Mean removal to separate mean and fluctuating components was done with a 120 s running mean. Longer running mean windows in most cases produced similar results, but were found in some cases to produce fluxes that diverged from the values calculated using shorter windows, perhaps indicating the influence of non-turbulent processes. Since shorter windows may exclude some slower turbulent eddies, 120 s was chosen as the longest acceptable window for mean removal. The fluctuating components of vertical velocity (w′) and concentration (c′) were multiplied to produce the instantaneous flux w′c′. Fluxes were calculated by averaging the w′c′ product in 30–60 min windows, chosen for sufficient averaging over statistical noise. To examine data with higher temporal resolution, cumulative fluxes were calculated by summing the w′c′ instantaneous flux for the duration of each deployment. For fluorescence at the Concord River site only, fluxes were calculated individually on manually identified segments of the time series corresponding to stable DOC values (see Section 3.3 below) using 60 s running mean windows that did not extend past the edge of each segment. Fluxes were then calculated for half hour flux windows by averaging w′c′ values for all available points in the flux window.

Power spectra were calculated using the pwelch function in MATLAB R2019b. Cospectra between velocity and concentration were calculated using the cpsd function, and summed from high to low frequencies to arrive at cumulative cospectra.

### 2.3. Seepage Rates, Temperature Profiles, Solar Radiation, Porewater Samples, TOC, Conductivity

Manual seepage meters as described by Lee [31] were used to independently measure inseepage of water through the sediment (i.e., submarine groundwater discharge). Meters were fabricated from the ends of 55-gallon steel drums, coated with epoxy paint and fitted with hose connections. Two meters were deployed at each site and their measured discharge rates were averaged.

To construct temperature profiles, rapid high-resolution temperature measurements were made in sediments and open water using a temperature probe built with a US Sensors GP104L8F thermistor located at the tip of a 5 cm long section of 2.5 mm OD, 1.8 mm ID stainless steel tube, which in turn was mounted on a telescoping series of stiffer tubing sections totaling 1.6 m in length; test leads were brought out the top of the tubing and the entire length of the assembly was potted with epoxy resin. Thermistor resistance was measured with a Tektronix DMM916 multimeter, and corresponding temperatures determined from a laboratory calibration curve. Readings typically settled to within 0.1 °C in 30 s or less. 

Solar radiation measurements were made with a LI-COR LI-185 photometer configured to measure photosynthetically active radiation (PAR, units of µmol/m^2^/s) with an upward-facing LI-192S (flat cosine response) sensor. 

Porewater samples were taken with a manual sampler fabricated from 316 stainless steel (1.27 cm OD, 2.1 mm wall thickness) and fitted with a pointed tip, behind which were cut a series of horizontal slots. Porewater entering the slots moved through an annular internal flow passageway into plastic tubing (1 mm ID, 3 mm OD) extending out the top of the sampler. Internal dead volume of the sampler, including the tubing, is approximately 3 mL. The sampler was driven into sediments by hand, and porewater was pumped using a 10 mL plastic syringe and Luer-Lok 3-port valve; the first 5 mL of sample were routinely discarded. Two porewater samples were obtained at each site.

Grab samples of stream or lake water were taken at each deployment site at the beginning and end of each field trial using combusted amber glass jars. Samples were stored for no more than 15 days at 2 °C. Porewater samples were filtered through combusted glass fiber filters. For TOC measurements, samples were acidified with 2N HCl and sparged for 15–20 min using TOC grade air, before measurement with an Elementar VarioTOC cube. Conductivity of surface grab samples and porewater samples was measured in test tubes at room temperature using an Amber Science Model 604 conductivity meter, compensated to 25 °C using the meter’s internal compensation. 

### 2.4. Heat Flux Measurement

Flux measured by eddy covariance is typically considered to directly represent flux to or from the sediment of an upstream footprint [32], potentially with some adjustment for changes within the water column between the sediment and the sensing volume [25]. The case differs for AEC-measured heat fluxes when solar heat input is significant. Solar radiation is another source of heat to the control volume that would, at steady state and in the absence of sediment flux, be balanced out by a (AEC-measurable) turbulent flux. 

Heat flux was derived by multiplying w′T′ values measured by AEC (where T is temperature) by the specific heat and density of water [3]. To provide a basis of comparison for heat fluxes measured by AEC, we constructed a conceptual heat budget for a control volume extending from the sediment to the sensing volume, comprising three major terms: (1) an eddy flux measured at the sensing volume; (2) a flux into or out of the sediment, driven by thermal conduction and/or porewater advection; and (3) a radiant solar heat flux downward into the control volume. Constraining the heat budget to these three terms requires several assumptions regarding horizontal homogeneity, horizontal transport, and storage, similar to those made for AEC flux measurements in general. In the present analysis, a storage term was not included because estimates of changes in heat storage based on the temperature record were found to create unrealistic and inconsistent contributions to heat balance. Thus, changes in temperature in the time series were attributed to the horizontal transport of water masses of different temperature, and any resulting effects on the heat budget through, e.g., differential advection [25] or variations in sediment conduction were neglected. 

To a first order, conservation then requires that the eddy flux of heat into or out of the control volume balances the sum of the heat flux at the sediment-water interface and the radiant solar heat flux input. 

Radiant solar heat flux was estimated for each flux window using downwelling PAR measurements taken manually during the deployments. PAR measurements (µmol/m^2^/s) were converted to radiant power density in the 400–700 nm range (W/m^2^) using the conversion factor of 4.57 µmol/m^2^/s per W/m^2^/s [33]. Measurements were taken just beneath the water surface to account for surface reflection, except at the Concord River site where measurement was taken on shore and then scaled for reflectance by 0.9, based on separate PAR measurements taken above and just below the surface on a different day. The solar fluxes were then corrected for attenuation using the Beer-Lambert law to the depth of the sensing volume, using an extinction coefficient determined for each site from underwater PAR profiles taken during the deployment. No correction was made for bottom reflection. For each deployment a depth-corrected solar flux measurement taken at a time when insolation was steady was extrapolated to the entire deployment by scaling by the continuous ambient light measurements made by the spectrofluorometer (i.e., relative photon count rates during the ‘LED off’ portions of the modulation cycle, measured at 465 nm), thus accounting for variations in insolation due to changing solar altitude and cloud clover. The final solar flux time series was integrated over each flux window. 

Heat flux at the sediment-water interface can include contributions from thermal conduction, analogous to a diffusive flux of solute, as well as advection from groundwater discharge where a temperature contrast exists between groundwater and surface water [3]. Conductive sediment heat flux was estimated from the observed temperature gradient in the sediment multiplied by a value of sediment thermal conductivity, taken for all sites as 0.0044 (cal/cm^2^/s)/(°C/cm) [34]. The advective contribution was estimated using the measured seepage rate, and the temperature of water immediately below the sediment surface as a proxy for groundwater temperature; at all sites, this heat flux was negligible compared to the conductive flux. Conductive sediment flux was calculated from a single temperature profile for each deployment, and is thus necessarily represented as constant for the entire deployment. 

### 2.5. Salt Flux Measurement

Conductivity fluxes were converted to dimensionally intuitive units of salt flux density (mg/cm^2^/h) by scaling by a commonly used approximate conversion of 0.64 mg/L salt per µS/cm [35]. AEC-derived salt fluxes were compared to fluxes expected based on groundwater discharge rates as measured by seepage meters (Section 2.3). For a known groundwater discharge rate, the expected benthic flux of salt due to groundwater discharge can be estimated from mass balance as [3]:

(1) $$F_{S}=\frac{q_{g}\left(S_{g}-S_{\omega }\right)\rho_{g}}{\rho_{\omega }}$$

where *F_S_* is the salt flux, *q_g_* is the groundwater flow, *S* is the mean salinity, *ρ* is the density (g/cm^3^), and the subscripts *g* and *ω* denote properties of the groundwater or surface water column. With the small salinity and temperature differences observed at our sites, we approximated density to be the same for groundwater and surface water. Thus, an independently estimated net flux of salt for comparison to AEC measurements was calculated as the groundwater discharge (inseepage) rate, as determined by seepage meters, multiplied by the salinity contrast, as determined by the conductivity (scaled to salinity) of surface grab samples and porewater samples.

The exact conversion of electrical conductivity to salinity depends on the specific ionic composition of waters, which varies considerably among freshwater systems and was not measured at these study sites. Here, fluxes derived from AEC and from seepage meters were both based on conductivity measurements, using the same scaling factor, and are thus directly comparable; however, absolute salt flux values contain uncertainty arising from the unmeasured ionic composition of the waters.

### 2.6. Field Deployments

**Figure 3:**
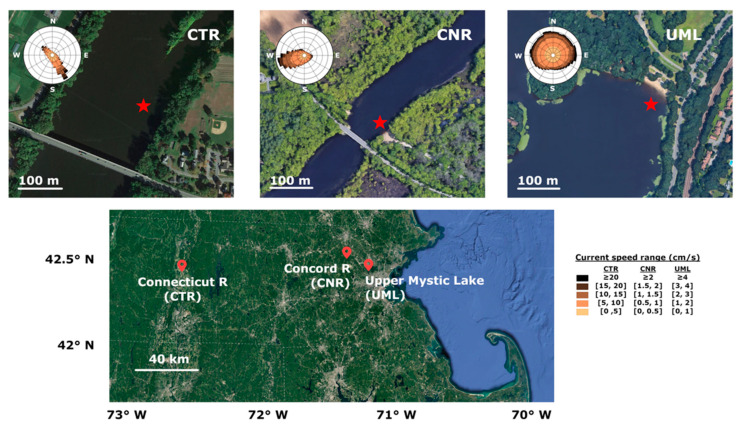
Location map of three field sites at which the eddy flux instrument was deployed, with current rose of horizontal water velocities measured during AEC deployment. Current roses indicate direction that current is traveling towards. Star indicates location of instrument deployment.

Field deployments were carried out at three different field sites to obtain a variety of physical and chemical conditions. The sites (Figure 3) include:

Connecticut River (CTR) near Sunderland Public Boat Ramp (Sunderland, MA, USA). In addition to the importance of this river as a regional water resource, the site was of interest because it provides a large expanse of relatively flat, gravelly bottom where flow appears to be steady and uniform. Although hydroelectric facilities modulate the flow of the river on a daily basis, river stage was constant during the period of deployment. 

At CTR, eddy fluxes were measured continuously for 3.5 h, beginning at 13:48 on 11 June 2021. Measurements were made approximately 16 m from shore at a water depth of 1.2 m. The sensing volume was located 16 cm above the sediment, as measured by the ADV’s boundary detection. Porewater samples, sediment temperature profiles, and groundwater seepage rates were measured within a few meters of shore.

Concord River (CNR) near Bedford Boat Ramp (Bedford, MA, USA). The bottom at this site is variously composed of a mixture of organic sediment, sandy material, rocks, and woody debris. Water velocity is lower than at CTR and flow appears less steady and uniform; shoreline projections as well as boating traffic appear to create back eddies at times. The area lies downstream of the extensive wetlands of the Great Meadows National Wildlife Refuge, and the water is humic-stained, enhancing fluorescence signals and attenuating sunlight.

At CNR, eddy fluxes were measured continuously for 4 h, beginning at 13:04 on 27 May 2021. Measurements were made approximately 10 m from shore, at a water depth of 1.6 m and measurement height of 11 cm above the sediment as indicated by the ADV’s boundary detection function.

Upper Mystic Lake (UML) near Shannon Beach (Winchester, MA, USA). Upper Mystic Lake has been previously described by, e.g., Varadharajan and Hemond [36]. Bottom sediments at the shallow experimental site are sandy with occasional small cobbles and plant detritus. Near-surface water movement appears to be influenced by wind drift, wave action, momentum input by the inflow of the Aberjona River, thermally driven density variations, and presumably by seiching as well. 

At UML, eddy fluxes were measured continuously for 3 h beginning at 14:26 on 4 May 2021. Measurements were made approximately 14 m from shore at a water depth of 0.8 m and measuring height 7 cm above the bottom, with the lander oriented to face approximately away from shore.

## 3. Results

### 3.1. Deployment Conditions

**Figure 4:**
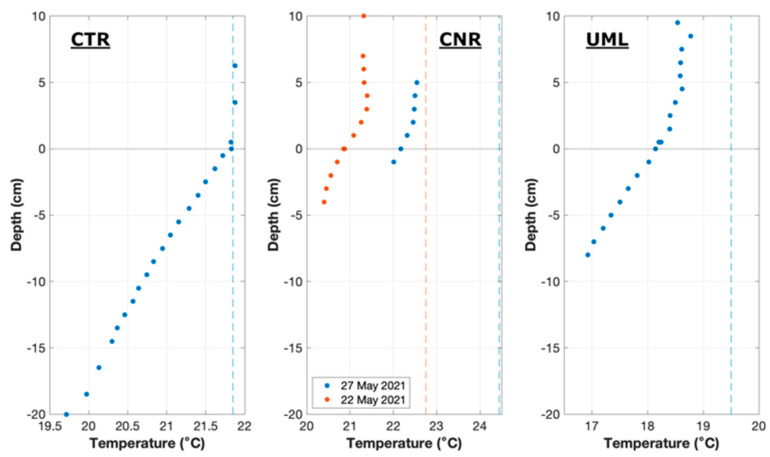
Temperature profiles at all sites. Surface temperatures are shown with dashed lines, and profile from a preliminary field trial at CNR is also shown for comparison. Sediment temperature gradients were estimated as 0.1 °C/cm at CTR, 0.15 °C/cm at CNR, and 0.17 °C/cm at UML.

At CTR, the ADV measured a pitch of −0.1° and a roll of 1.7°, indicating a slightly sloped sediment floor. Measured current velocities averaged 7 cm/s in magnitude and were mostly oriented diagonally toward shore, slightly rotated from the expected downstream direction of the river, possibly due to hydrodynamics relating to the upstream island. The river was well-mixed vertically at this location, with water temperature vertically homogeneous except for a very thin bottom layer (Figure 4). 

At CNR, the sediment floor at the measurement location was sloped towards the center of the river. The instrument was oriented to face into the direction of river flow, resulting in a tilt measured by the ADV as 1.8° pitch (forward/back) and 7.2° roll (side to side). Current velocities at the measuring site had an average magnitude of 0.9 cm/s with a component directed away from shore. The water column appeared to be weakly stratified thermally, with near-surface waters being 1 to 2 °C warmer than near-bottom waters. 

At UML, the ADV measured a pitch of −1° and a slope roll of −0.5°. Wind-driven waves were observed at a frequency on the order of one Hz. Average water velocity magnitude was 1.7 cm/s, and direction was variable.

The lander was designed to avoid measurement interference by the lander legs under conditions of known, unidirectional flow. However, at our sites, the observed current directions were not unidirectional and sometimes not in the expected direction (Figure 3). Although Reimers et al. [37] found interference by their lander frame to be relatively minor relative to (wave-driven) background turbulence in flume experiments, such interference may have greater impact relative to the lower turbulence at our sites. In the future, expanded reconnaissance to identify and characterize sites with simpler hydrodynamics would avoid any uncertainty due to possible lander interference.

### 3.2. Velocity Data Quality

At CNR and UML, ADV data were screened with a 70% correlation threshold, resulting in <0.3% of data removed. At CTR, ADV correlations were somewhat lower, evidently because the combination of measurement height (16 cm) and bottom characteristics resulted in interference between the ping pairs of the ADV (‘weak spot’). The interference was not apparent in pre-deployment checks closer to shore due to different bottom characteristics, which can affect the location of the weak spot and/or the apparent boundary location seen by the ADV [28]. Thus, a lower correlation threshold of 60% was used in screening the velocity data from CTR, resulting in 19% of the data replaced by linear interpolation. More stringent correlation screening was found to exclude a large amount of data (e.g., 45% data excluded when using a correlation threshold of 70%) and result in generally lower fluxes, as would be expected if eddies in the velocity signal were omitted. A correlation threshold of 60% was found to produce similar results to lower thresholds. 

### 3.3. Concentration Measurements

**Figure 5:**
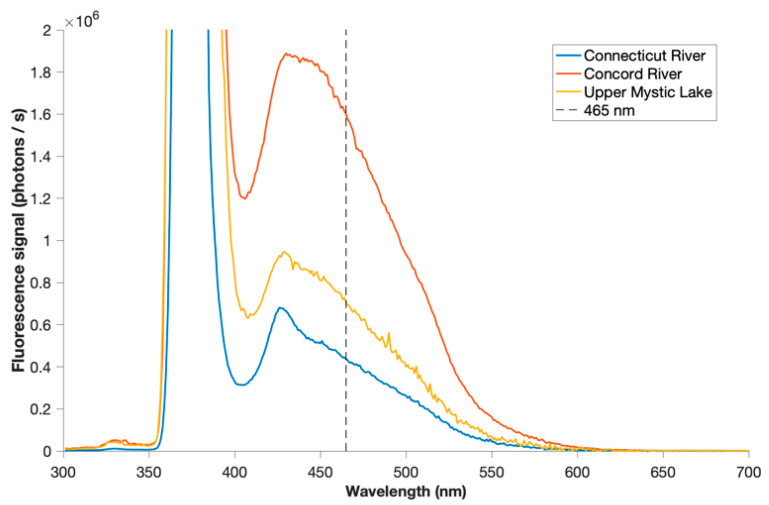
Fluorescence emission spectra measured by FACT sensor at all three sites, including LED excitation scattering at 375 nm. Emission was measured at 465 nm for eddy covariance trials.

Fluorescence measured at all three sites had a consistent emission spectrum when excited at 375 nm (Figure 5). At CNR, occasional coincident pulses of a few seconds or less were observed in the fluorescence, velocity, and velocity amplitude time series. These transient events were hypothesized to arise from floating detritus or other particulate material, and were manually identified and removed from both DOC and velocity time series. Occasional pulses in fluorescence alone were also observed and removed. In addition, at several points in the record the fluorescence baseline was observed to shift by several tenths of a mg/L, often in less than a minute (Figure S5). Similar shifts were also observed during earlier, preliminary tests at CNR but never at any other site or in laboratory tests. These shifts are thought to arise from varying flow patterns at river scale (e.g., large eddies or internal waves in the weakly stratified water column). Because advection-driven change in DOC storage in the water column could introduce ambiguity into the computation of eddy fluxes, fluxes for fluorescence at this site only were calculated individually on plateaued segments of the time series, and 35 min of the record is omitted. Consistent with the hypothesis of weak stratification, conductivity and temperature time series at this site also exhibited coinciding excursions of lower temperature and higher conductivity, possibly reflecting eddy transport of a slightly cooler and saltier near-bottom layer (Figure S5). 

At CTR, occasional positive spikes were observed in the conductivity signal. There was, again, no evidence of sensor malfunction, and a considerable history of measured conductivity data that was free of such spikes. These spikes are thought to result from the passage of detrital material through the sensing volume, and were manually identified and removed. 

### 3.4. Eddy Fluxes of Salinity 

**Figure 6:**
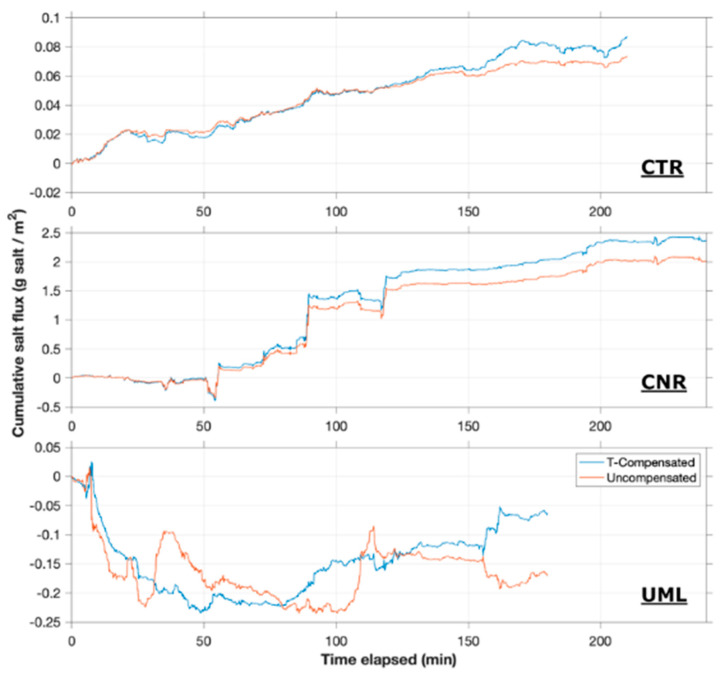
Cumulative eddy flux of salt at all sites. Blue traces are as calculated with temperature compensation, while red traces are fluxes based on raw electrical conductivity data.

Cumulative eddy fluxes of salt for each of the deployments are shown in Figure 6. Except for the first hour at UML, a consistent positive (upward from sediment to water column) benthic flux of salt was observed at all sites.

At CTR, groundwater discharge was estimated by the seepage meters to be upward at 7.2 cm/day. Conductivity of porewater samples averaged 320 µS/cm, while river water averaged 150 µS/cm, resulting in an estimated salt flux of +0.3 g/m^2^/h. In comparison, the AEC instrument measured a cumulative eddy flux of +0.09 g/m^2^ upward over the 3.5-h deployment, thus averaging 0.03 g/m^2^/h. Importantly, measured AEC flux was monotonic in the expected upward direction, resulting in a steady increase of cumulative benthic salt input to the river, as is expected under steady hydraulic conditions. 

At CNR, seepage meters measured a groundwater discharge rate averaging 8.6 cm/day. Conductivity of porewater samples averaged 604 µS/cm, while river water averaged 520 µS/cm, resulting in a calculated upward salt flux of +0.15 g/m^2^/h. In comparison, the cumulative eddy flux measured by the AEC instrument over four hours was approximately 2.5 g/m^2^, or an average of 0.6 g/m^2^/h. Importantly, the measured eddy flux was again consistently positive throughout the deployment period.

At UML, groundwater discharge rate measured by seepage meters was 1.4 cm/day upward during the fall of 2020. Conductivity of porewater samples during the spring of 2021 averaged 930 µS/cm, while lake water averaged 740 µS/cm. Recognizing uncertainty due to non-contemporaneous measurements of seepage rate and porewater conductivity, the best estimate of salt flux is of the order of +0.07 g/m^2^/h. The eddy flux of salt measured by AEC was negative during the first hour of deployment; for the remaining two hours of deployment, a cumulative upward flux was measured of approximately 0.15 g/m^2^, averaging 0.07–0.08 g/m^2^/h.

### 3.5. Eddy Fluxes of Heat

**Figure 7:**
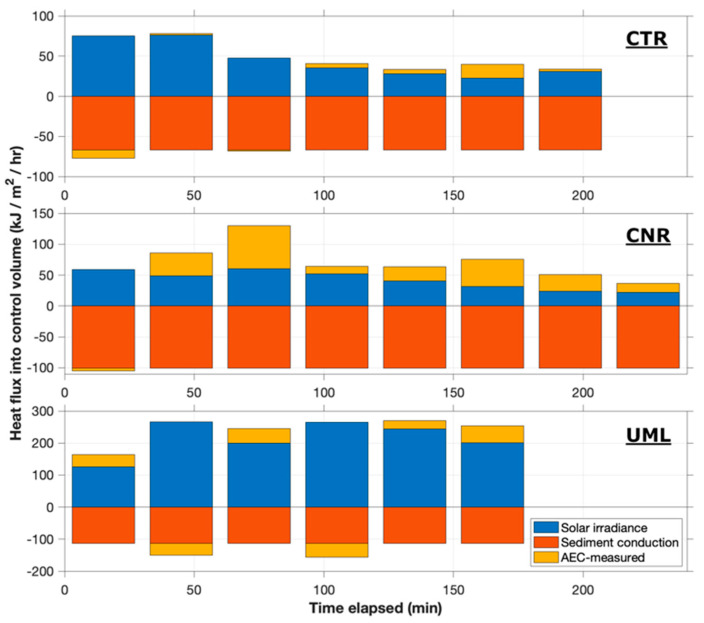
Benthic heat flux balance at all sites. Positive heat fluxes represent input to the control volume (downward solar irradiance or downward eddy flux at the sensing volume), while negative fluxes represent heat leaving the volume (downward conduction at the sediment-water interface or upward eddy flux at the sensing volume).

The balance between the solar, sediment, and AEC-measured eddy heat fluxes at all sites is shown in Figure 7. Positive heat fluxes represent input to the control volume (downward solar irradiance or downward eddy flux at the sensing volume), while negative fluxes represent heat leaving the volume (downward conduction at the sediment-water interface or upward eddy flux at the sensing volume). By energy conservation, at steady state, the summation of positive and negative bars in this figure should equal zero. 

At all sites, conductive sediment heat flux inferred from temperature gradients in the sediment was downward. At CTR, the estimated sediment flux was 66 KJ/m^2^/h, which was similar in magnitude to the solar input during the first two flux windows (about 75 kJ/m^2^/h). During this period, the vertical eddy fluxes were small and variable, consistent with the solar and sediment fluxes being nearly in balance. As solar input decreased during the afternoon, the downward eddy flux of heat (represented as positive in Figure 7) began to increase in magnitude. The consistent downward eddy flux in the afternoon acted in a direction to balance the total heat flux in the control volume against heat loss into the sediment. 

At CNR, the conductive flux into the sediment was estimated to be approximately 100 KJ/m^2^/h. Solar heat input was lower than at CTR or UML due to the greater light attenuation in the water column (extinction coefficient α = 1.87/m, compared to 0.77/m at CTR and 1.2/m at UML), but was nonetheless significant during the deployment due to high solar altitude and minimal cloud cover. The solar heat flux ranged as high as 60 KJ/m^2^/h, decreasing to 20–30 KJ/m^2^/h later in the afternoon. Except for the first window, the eddy fluxes of heat were consistently downward, contributing to closure of the energy balance. 

At UML, the estimated conductive flux into the sediment was approximately 120 KJ/m^2^/h. The relative importance of the solar flux was high at this location and time due to the high solar altitude and relatively shallow water. Thus, solar fluxes were the dominant input to the sediment, averaging 220 KJ/m^2^/h. The eddy heat flux was much smaller, ranging from 26 to 53 KJ/m^2^/h, and fluctuating in both magnitude and direction.

### 3.6. Eddy Fluxes of DOC

**Figure 8:**
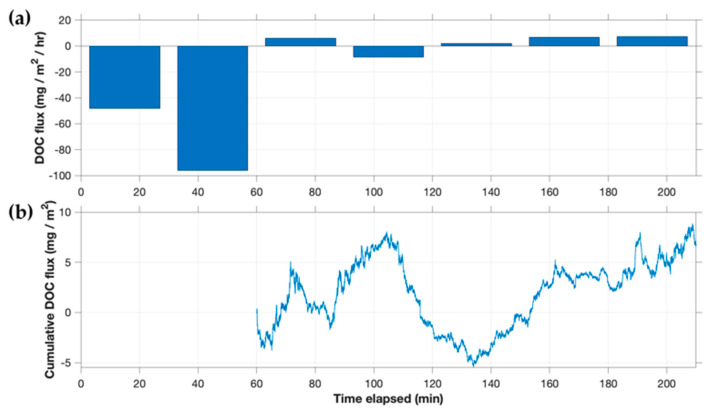
(**a**) DOC flux and (**b**) cumulative flux at the Connecticut River site (CTR).

Fluorescent DOC fluxes measured by AEC at CTR are summarized in Figure 8. During the first 45 min, large and varying fluxes were observed, which also coincided with the period of maximum insolation (Figure S4). Due to potential contamination by ambient light (discussed further in Section 4.3.1), these fluxes are omitted from Figure 8b. After this initial period, fluxes were much smaller (of the order of 2–8 mg/m^2^/h) and generally positive. Porewater concentrations of DOC at this site (3.02 mg/L and 4.22 mg/L for duplicate points) were similar to surface water concentrations (3.91 mg/L and 3.93 mg/L at the start and end of the AEC trial, respectively). These values suggest that any benthic DOC flux due to groundwater inseepage or molecular diffusion was likely minimal. 

**Figure 9:**
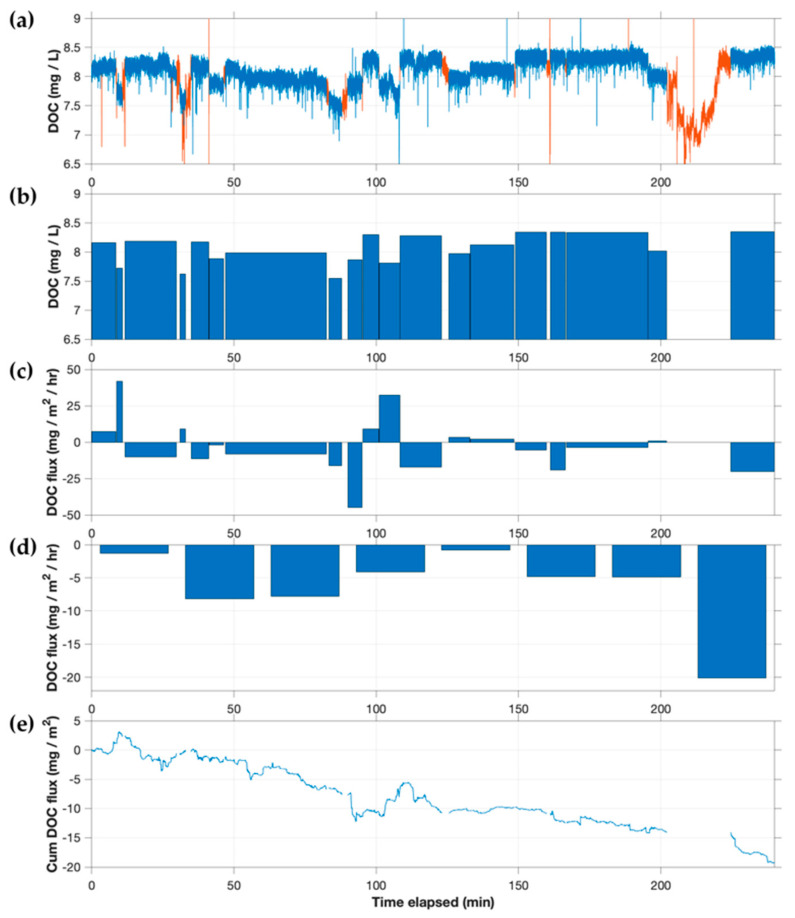
DOC and DOC flux at the Concord River site (CNR). (**a**) DOC time series showing omitted sections in red. (**b**) Manually identified ‘plateaus’ of DOC concentration in time series, shown as average DOC value for each plateau. (**c**) DOC flux calculated individually for each plateau. (**d**) DOC flux calculated in 30 min flux windows by averaging all available w′c′ values in each window. (**e**) Cumulative DOC flux.

Eddy fluxes of fluorescent DOC measured at CNR (Figure 9) were consistently downward (negative) and of the order of 1–8 mg/m^2^/h, except during the last 15 min of deployment when a significantly larger downward flux (20 mg/m^2^/h) was measured. Similar to CTR, the porewater concentration of DOC (7.88 mg/L and 8.22 mg/L for duplicate points) was similar to surface water (8.11 mg/L and 8.34 mg/L at the start and end of the AEC trial, respectively), and thus neither groundwater seepage nor molecular diffusion appears to be an important contributor to benthic DOC flux.

**Figure 10:**
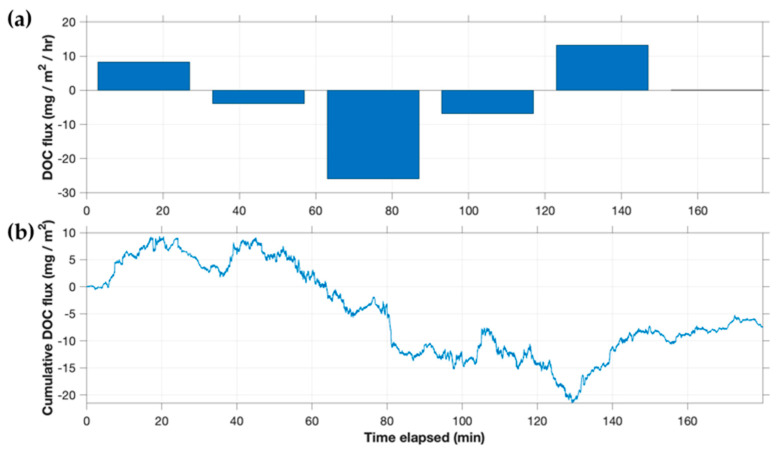
(**a**) DOC flux and (**b**) cumulative flux at Upper Mystic Lake.

At UML, the AEC instrument measured both positive and negative benthic eddy fluxes of DOC, ranging from −16 to + 7 mg/m^2^/h, with an overall average of −2.5 mg/m^2^/h (Figure 10). At this site, the concentration of DOC measured in porewater (3.17 mg/L and 4.52 mg/L for duplicate points) was lower than that in surface water (5.01 mg/L and 5.20 mg/L at the start and end of the AEC trial, respectively). Given the groundwater inseepage rate observed from seepage meters, and using an approximate DOC differential of 1.3 mg/L between porewater and surface water, a net apparent downward eddy flux of DOC due to groundwater could be expected on the order of 0.8 mg/m^2^/h. Downward flux due to molecular diffusion, estimated using the depth of the well points (6–13 cm) to construct a two-point gradient and a diffusion coefficient on the order of 10^−6^ cm^2^/s [14], was found to be negligible (<0.01 mg/m^2^/h). Thus, the measured eddy flux is in the direction expected, but of larger magnitude. However, high ambient light during the UML deployment (Figure S6) may have increased noise in the measured AEC flux, as discussed further in Section 4.3.1.

## 4. Discussion

### 4.1. Salt Fluxes

Because groundwater discharge from the sediments occurred at all three field sites, and groundwater conductivity contrasted sufficiently with surface water conductivity, the salt fluxes measured by AEC could be compared directly with independent estimates made with seepage meters together with electrical conductivity. Salt fluxes measured by AEC were generally steady and of the same sign and order of magnitude as expected from these independent measurements. Exact agreement was not expected, as groundwater discharge is typically very heterogeneous [16,38], and only two seepage meters were deployed in the present study.

At CTR, the flux measured by AEC was steady and upward throughout the deployment. Although smaller than that inferred from the seepage meters by around a factor of 10, the seepage meters were necessarily deployed significantly closer to shore than the AEC instrument; given typical groundwater flow paths, inseepage toward the center of the river was expected to be less than near the banks [39]. Spatial heterogeneity in the groundwater discharge was also visually evident from the patchiness of iron precipitation (often associated with groundwater seepage) observed along exposed nearshore sediments (Figure S3).

The benthic eddy flux of salt at CNR was also upward and steady, but at this site was larger than that estimated from seepage meter measurements by a factor of approximately 4. Given the known heterogeneity of groundwater, and the fact that only two seepage meters were deployed, this amount of difference is not unexpected.

Benthic flux of salt measured at UML was negative during the first hour of deployment, but became positive for the remainder of the three-hour deployment. One hypothesis for this variability is a shifting footprint (Figure 3); however, this hypothesis could not be tested with only two seepage meters. Another hypothesis is artifact introduced by the wind-driven waves. A local peak at wave frequency was observed in the power spectra of both conductivity and vertical velocity signals during the first hour, and cumulative cospectra calculated in 15 min windows showed contributions at wave frequency throughout the first hour and a half of deployment (Figure S7). Wave-induced turbulence can contribute to transport by eddy diffusion, but would be expected to give rise to apparent eddy fluxes in all three channels; however, no clear components at wave frequency were observed in the cospectra of temperature or fluorescence after the first 15 min of deployment. Consequently, the measured downward eddy flux of salt during the first hour is thought most likely to be an artifact of wave action, and specifically related to the flexing of the cables to the conductivity cell by water movement. Later in the deployment, when wave action was less, the upward eddy flux of salt at UML measured by AEC was steady and coincided well with the value estimated from seepage meters. Based on these observations, for the CTR and CNR deployments, additional zip ties were employed to fasten all cables more tightly to the lander to minimize possible cable motion. 

Salt flux data also highlight the importance of accurate compensation of conductivity for temperature changes, which had a small but distinct impact on calculated fluxes (Figure 6). The observed temperature fluctuations, which were correlated with vertical velocity where a net heat flux existed, on occasion translated to fluctuations in electrical conductivity that were of similar magnitude to those contributing to salt flux. Because small fluctuations can be significant in AEC calculations, it is particularly important to use an accurate temperature compensation algorithm together with a temperature sensor with a similar response time and sensing volume as the conductivity cell [40]. 

In summary, AEC measurements of salt fluxes agreed, within reasonable error limits, with independent measurements made by seepage meter and conductivity measurements. The ability to measure benthic fluxes of salt by AEC is itself a potentially powerful tool at locations where groundwater inseepage is responsible for the benthic fluxes of other substances of interest, such as trace metals, nutrients, and pollutants; salt may serve as a tracer for groundwater and thus in some situations enable determination of the fluxes of these additional substances. AEC is an attractive alternative to traditional seepage meters, allowing integration over larger footprints as well as, in the case of non-automated seepage meters, finer time resolution over longer time periods. Finally, the observation of a usable salinity contrast between surface and groundwater at each of three arbitrarily chosen sites in this study suggests that AEC measurements of benthic salt flux, and the use of such flux as a geochemical tracer, may be feasible at many locations even in freshwater systems.

### 4.2. Heat Fluxes

The validity of heat fluxes measured by AEC is supported by their general agreement with the heat balances inferred from independent determinations of solar and sediment heat fluxes (Figure 7). Despite the several approximations that were necessary when making comparison between eddy heat fluxes and total benthic heat budgets, upward and downward fluxes were generally balanced within reasonable bounds of uncertainty. 

Uncertainty in the estimates of heat conduction into the sediment arises primarily from uncertainty in the estimate of sediment thermal conductivity, whose direct measurement was not feasible in the present study. Although a value typical of freshwater sediment was used, sediment thermal conductivity is known to vary somewhat depending on various sediment properties [34]. Error associated with the measurement of in-sediment temperature profiles appears to be minimal, as thermistor readings were stable, and scatter in the sediment temperature profiles was negligible. 

The estimation of solar heat fluxes on the basis of photometric measurements also contains significant but difficult-to-quantify uncertainty; absent data on the spectral distribution of radiant energy, conversion of PAR to radiant solar heat fluxes was based on conversion factors taken from the literature.

Given these uncertainties, the extent to which estimated heat fluxes were in reasonable balance at each site is encouraging. In addition, comparison of the heat balances among deployment sites illustrates trends that are consistent with expectation. In the relatively clearer waters of the Connecticut River, the total heat flux toward the sediment was dominated by solar input, and during the period of highest insolation an upward eddy flux of heat was actually observed, consistent with some degree of net solar heating of the sediment surface. By contrast, in the humic-stained waters of the Concord River, the solar flux reaching the benthic control volume was smaller. In compensation the eddy heat flux was relatively larger and consistently downward, becoming the dominant heat source to the sediments during several flux windows. At the shallower Upper Mystic Lake site, with relatively clear water, solar flux to the sediment was large, while sediment conduction was also larger than at other sites. Consistent with this observation, AEC-measured heat fluxes were small and varying in direction. In general, while there are insufficient data to construct rigorous error budgets, the heat fluxes measured here by AEC are consistent with expectation and support the ability of the FACT-based AEC system to measure these fluxes.

### 4.3. DOC Fluxes 

In contrast to salt and heat fluxes, obtaining measurements of benthic DOC flux that are independent of AEC is notably difficult. Flux chambers, incubations, calculations from sediment microprofiles, and whole ecosystem mass balances all have large sources of uncertainty or are highly demanding logistically. As a result, assessment of the validity of DOC fluxes observed in this study relies in part on inter-channel comparisons, enabled by the fact that the three fluxes measured by AEC share a common velocity record and use the same signal processing algorithms. Specifically, the fact that independent data support the validity of salt and heat fluxes, and that fluorescence sensor performance has been independently verified, are taken as supporting the validity of the DOC fluxes measured by AEC. However, even given near-ideal behavior of the fast spectrofluorometer as a photon counter, two additional sources of possible artifact—ambient light interference and variability of the fluorescent properties of DOC—deserve discussion. 

#### 4.3.1. Ambient Light Effects 

To reject ambient light, the FACT sensor employs a modulation technique combined with a sensitive photon counting detector [8]. This technique proved sufficient for rejecting bulk ambient light features; for example, at Upper Mystic Lake, a signal in ambient light at wave frequency was not seen in the modulated difference signal (Figure S6), and its removal was corroborated by power spectra (Figure S8).

Nevertheless, high ambient light levels present certain fundamental limits to the precision of fluorescence measurements, in part because statistical photon counting errors are proportional to total counts. Thus, although the ambient light signal is subtracted from the total fluorescence signal, its noise still propagates. The modification of the FACT sensor, prior to these field studies, to incorporate a more powerful excitation source reduced the impact of this noise by increasing the ratio of the fluorescence signal relative to ambient light. High ambient light levels can also reduce precision due to the dead time effect intrinsic to photon counters, which requires corrections as detectors approach within an order of magnitude of saturation (10^7^ to 10^8^ photons/s for FACT) [41].

At Upper Mystic Lake, high ambient light levels throughout the deployment resulted, as expected, in increased noise in the DOC signal (Figure S6). Noise levels that rival or even exceed actual fluctuations, a particular problem with small fluxes, are acceptable for AEC if the noise is randomly distributed around the mean [42]. However, a high level of noise, even when random, can translate to larger transient calculated fluxes in both directions (essentially, noisy fluxes). Thus, a longer averaging period of 60 min was used for DOC fluxes at UML (Figure 10). A one-hour resolution is a relatively fine temporal resolution compared to traditional methods, although it may be too long to capture some dynamic changes expected in benthic fluxes, especially at sites with permeable sediments [2,43]. In general, however, results at UML were consistent with the predicted direction (downward, because porewater concentrations were lower than lake water concentrations) though larger by a factor of about three than would be expected on the basis of groundwater inseepage. Microbial consumption of DOC is potentially another factor contributing to this observed result, as discussed further in relation to CNR data.

Ambient light was also high, particularly during the first hour, during the CTR deployment, when brief (lasting several seconds) ‘pulses’ in ambient light were seen that appeared to be overcorrected by the modulation feature. The resulting brief decreases in the DOC signal are suspected to have created substantial negative contributions to flux (Figure 8a and Figure S4). This overcorrection did not appear to be due to a dead time effect in the photon counter, as the application of a dead time correction [41] did not remove the short decreases from the time series and actually increased the magnitude of the calculated DOC flux. These ambient light pulses were not observed at any other site, appeared to only exist during periods of stronger ambient light at CTR, and did not have a notable spectral signature. As such, we do not believe that they are due to instrument artifact, and instead hypothesize that they are related to refraction of solar light by surface ripples. As a result, we lack confidence in the DOC flux data from the first hour of the CTR deployment (which are therefore omitted in Figure 8b), and data from the second hour may be partially contaminated as well. During the third hour, with lower solar altitudes later in the day, the measured benthic DOC flux was steady and monotonic upward. 

By contrast, at CNR, high light attenuation in the humic-stained waters resulted in ambient light being an order of magnitude smaller than the fluorescence signal (Figure S5). Any potential effect of ambient light on AEC measurements at CNR thus appears to be negligible.

The impact of ambient light on the quality and reliability of fluorescence fluxes is ultimately a signal-to-noise issue that depends on various interacting factors related to both the signal (concentrations and gradients of fluorescent material, integration time, flux window length, excitation power, etc) and noise (the amount of ambient light, which is dependent on time of day, cloud cover, water column attenuation coefficient, depth, etc). Deployment of FACT-based AEC under conditions of clear waters or sunny days is not necessarily precluded, but may require careful evaluation and consideration in data processing.

#### 4.3.2. FDOM vs. DOC 

Intrinsic to the use of fluorescence as a measure of DOC is the assumption that the composition of DOC is the same in all measurements, including those from which a fluorescence-to-concentration calibration is derived. DOC is in fact a heterogeneous mix of compounds whose composition varies over both space and time. The FACT sensor (375 nm excitation, and an emission wavelength of 465 nm chosen at the peak of observed fluorescence spectra (Figure 5)) targets the humic fraction of FDOM most closely aligned with peak ‘C’ as classified by Coble [44]. Relative to other operationally defined fractions, this fraction has been associated with more aromaticity, more conjugation, and higher molecular weight [45], and is generally considered more refractory [11,46], in contrast with protein-like fractions that have been associated with higher bioavailability [47]. 

In the present study, the fluorescence-concentration calibration was consistent for all surface water samples (Figure 2), giving some confidence in its general applicability in this region and season. However, preferential consumption or release of one fraction relative to others may affect the applicability of this calibration to benthic DOC fluxes, particularly at CNR where DOC consumption appears to be important. In other studies, benthic fluxes of different fractions of FDOM and DOC have been measured separately, recognizing that their proportions may vary [11,12,48]. Future modifications of the FACT sensor could include different excitation wavelengths (e.g., 220–280 nm [11,45,49]), enabling measurement of fluxes of different fractions of DOC. In addition, characterizing the fluorescence-concentration calibration of porewaters in future studies would provide further information regarding the uncertainty associated with assuming a single calibration of fluorescence to DOC concentrations. Because the FACT spectrofluorometer is an open-path device that requires larger sample volumes (>500 mL) to avoid interference from container walls, but collection of large volumes of sediment porewater is not practical, this task will likely require use of a closed-path spectrofluorometer whose characteristics can be matched to those of the open-path FACT sensor.

#### 4.3.3. A Biotic Sink for DOC at the Concord River

Benthic cycling of DOC is a complicated interplay of abiotic and biotic processes, including diffusion between water column and sediment porewaters, groundwater input, sedimentation or sediment resuspension, adsorption or desorption, leachate from terrestrial detritus, exudates from primary producers like algae and macrophytes, and processing and consumption by heterotrophic bacteria for respiration and growth [13,15,50]. In streams and other freshwater systems, detritus-based pathways are particularly important to organic matter cycling, as organic carbon originating from allochthonous and autochthonous sources is processed along multiple pathways, transformed between particulate and dissolved fractions, and ultimately buried in sediments, transported downstream, or respired to CO_2_ [50]. Thus, the sediment can be both a source and a sink of DOC.

There are relatively few direct measurements of benthic DOC flux in freshwater systems, but several studies across coastal and freshwater ecosystems indicate that the sediment is often a source of DOC and/or FDOM to the overlying waters, driven mainly by higher concentrations found in porewaters relative to surface waters [11,12,13,15,46,48,51]. At the sites in the present study, however, a comparable sediment efflux may not a priori be expected; consistent with some other streams [52], DOC concentrations in porewaters were not elevated. Instead, mechanisms such as microbial consumption within sediments may be more important drivers of benthic flux, resulting in the consistent downward flux observed at the Concord River site (CNR). Sediment consumption has been inferred to affect directly measured fluxes elsewhere; in a mangrove forest, no net efflux of DOC was observed with benthic chambers despite significantly elevated porewater concentrations, attributed to particularly high bacterial activity in the sediment fueled by DOC generated deeper within the sediment [53]. 

In stream ecosystems, studies have documented significant consumption of DOC by hyporheic sediments, based on observations of decreasing DOC concentrations and bacterial activity along hyporheic flowpaths, as well as on the basis of incubation and mesocosm experiments. In these systems, active exchange between surface waters and hyporheic porewaters provides a renewable source of DOC and nutrients to fuel sediment microbial activity [52,54,55]. Such dynamics, however, may be especially problematic for benthic chambers and core incubation technique to capture; with the most labile DOC fractions likely respired on the scale of minutes [56], these methods are unable to simulate the regular supply of external DOC that fuels sediment metabolism in these settings. 

DOC addition experiments in streams, involving in situ steady state or pulsed additions of a chosen form of DOC (often simple compounds or leachates), have been used to quantify sediment uptake in terms of uptake velocities [57]. In general, DOC uptake rates are highly variable and appear to depend on many factors, including the amount of exchange with, and size of, the hyporheic zone, nutrient availability, the rate of microbial activity (itself a function of various environmental conditions), and, to a large degree, the bioavailability of the DOC, which can vary significantly among systems [50,52,55,57,58]. 

In context of these DOC studies, the consistent downward benthic flux of DOC observed by AEC at CNR appears to be reasonable. The corresponding uptake velocity on the order of 0.01 mm/min is well within the ranges compiled by Mineau et al. [57] of 0.002 to 7 mm/min for leaf leachates, or 0.0005 to 1.7 mm/min with an estimated scaling to the bioavailability of ambient DOC. 

Of the three sites investigated in this study, CNR has the highest background concentration of DOC (Figure 2), perhaps promoting the development of sedimentary biota that utilize this DOC as an energy resource. As DOC concentrations generally decrease longitudinally down river networks, and lability likely decreases as well [52,58], the proximity of the CNR measurement site to upstream wetlands (which can represent an important source of bioavailable carbon to downstream systems [47,55,59]) further support this interpretation. 

## 5. Conclusions

Field tests of the FACT sensor as part of an aquatic eddy covariance instrument produced estimates of benthic fluxes of fluorescent material, salt, and heat at three distinct freshwater sites. Benthic fluxes of salt estimated by AEC were consistent with independent measurements of groundwater discharge made with seepage meters, considering the typical heterogeneity of groundwater flow patterns. Fluxes of heat were generally consistent with the balance of incoming solar radiation and downward conduction at the sediment surface. Benthic fluxes of fluorescing substances, whose measurement was enabled by the fast spectrophotometer channel, were used to infer fluxes of dissolved organic carbon. Results revealed the potential importance of sediment DOC uptake to benthic fluxes, and the unique capability of AEC to measure such fluxes. The ability to measure DOC fluxes by AEC is a useful tool for gaining insight into biogeochemical processes; more generally, ability to measure fluxes of fluorescent materials may assist in contaminant fate and transport studies. 

### 5.1. Synergism among Sensor Channels

Several synergisms were recognized when using the FACT three-channel sensor. Firstly, having multiple channels of contemporaneous and co-located sensor data assisted in assessing data quality. For example, validation of salt and heat flux data obtained by AEC supported the validity of AEC measurements of DOC flux, as all three channels share a common velocity record and use the same signal processing techniques. Comparison between the channels also assisted in attributing signal features such as perturbations by particles and wave artifacts. 

An important synergism exists between the temperature and electrical conductivity channels, as the fast temperature channel proved critical for correcting electrical conductivity signals for temperature effects (Figure 6).

A third cross-channel synergism arose from the ability of the spectrofluorometer channel to measure rapidly fluctuating solar radiation in the water column (Figures S4–S6), and thereby obtain integrated measures of solar heat flux under variable cloud cover. This obviated the need for deployment of a separate continuously recording solar radiation meter. 

### 5.2. Tracer Techniques for other Biogeochemicals and Pollutants

Knowledge of benthic fluxes is an important and often-lacking component of many studies, whether of natural element cycling or of pollutant fate and transport. However, sensors are not yet available to measure many benthic fluxes of interest. In settings where the benthic flux of such substances is driven by the flow of groundwater into surface water, a benthic salinity flux may prove to be a valuable tracer. Data from the three field sites in this study suggest that salinity contrasts between sediment porewater and surface waters may often be adequate to enable measurement of groundwater inflow to surface waters even in many, if not most, freshwater systems. FDOM may also be a potential tracer for groundwater discharge in some systems, as FDOM signatures may be usable for distinguishing groundwater of different sources from ambient surface waters [60]. 

The conductivity channel may be especially conducive to an investigation of heterogeneity, as measurements by seepage meters along with porewater conductivity measurements provide relatively reliable, conservative estimates of benthic salt flux. Thus, deploying several replicates of chambers in the vicinity of an AEC setup, similar to Attard et al. [61], may provide some estimate of the heterogeneity of groundwater at a site and the ability of the AEC system as deployed to average over this heterogeneity.

### 5.3. DOC Fluxes, and the Utility of a Fast Spectrofluorometric Sensor

Benthic DOC fluxes are important in understanding both ecosystem function and carbon cycling. Net benthic DOC flux can occur in either direction, with biota at the sediment-water interface playing a key role. Measurement of benthic DOC fluxes is challenging with other techniques like benthic chambers and core incubations, due to their potential alteration by the techniques themselves. AEC avoids many of these liabilities and is able to provide benthic flux data with relatively high (hour or less) temporal resolution. DOC-based AEC may be especially useful where DOC consumption occurs at the sediment-water interface, as appears to occur at the Concord River site, but is equally valuable where DOC efflux is expected to occur, such as sites with anoxic sediments and/or iron hydroxide dynamics [12,13,46], or over phytobenthic communities like seagrass meadows, macroalgae, and coral reefs, where DOC is exuded by the primary producers [62,63,64,65]. 

### 5.4. Instrument Development

There appears to be an excellent opportunity to use AEC to simultaneously measure benthic DOC uptake and benthic O_2_ uptake, by adding an appropriate fast oxygen sensor to the instrument (e.g., [17]). The ability to make such measurements with minimal disturbance, and obtain continuous records over one or more diurnal cycles, could make it possible to test a variety of hypotheses regarding the functioning of benthic and hyporheic communities. 

Several opportunities exist to enhance performance of the FACT spectrofluorometer. Notably, taking advantage of the tunable monochromator and the PMT’s UV sensitivity, potential installation of a shorter excitation wavelength (e.g., 220–280 nm) can be used to target additional fractions of FDOM [11,45,49]. This would enable further and more detailed studies of FDOM dynamics, as well as specific applications such as targeting the amino acid-like FDOM fractions that distinguish coral exudates from algal exudates [64]. In addition, shorter excitation wavelengths can potentially be used to target fluorescing pollutants in sediment contamination studies; for example, Wasswa et al. [66] found significant correlations between tyrosine-like and tryptophan-like FDOM fluorescence peaks (excitation ~275 nm) and various fluorescing organic pollutants. Similarly, Sinfield et al. [67] demonstrated use of a far-UV Q-switched laser as an excitation source in the detection of hydrocarbon contaminants by fluorescence. 

However, shorter wavelengths in light sources generally accompany tradeoffs with optical power, beam quality, transmissibility through optical fibers, power consumption, heat generation, size, and cost. The installation of a shorter wavelength source may become more attractive and/or feasible as optical device technology continues to improve, or in certain environmental conditions. For example, if lower ambient light is expected (as was seen at the Concord River site), optical power and transmission may be less important for achieving an adequate signal to noise ratio; or if turbulent transport is dominated by slower eddies, slower measurements and longer integration times may be used, enabling higher signal to noise ratios. Sensitivity can also be increased with the use of broader wavelength bands for the excitation source or detector (e.g., by use of an optical filter rather than the monochromator), with tradeoffs in specificity of target compounds [66].

In summary, opportunities for further advances include both enhancement of spectrofluorometric capabilities, and further exploitation of synergism resulting from acquisition of multiple channels of concentration data. Important applications include both monitoring of heavily contaminated sites for public health purposes, and basic biogeochemical research.

## Data Availability

Data from this study can be found at https://fairdomhub.org/studies/1083, uploaded 6 September 2022.

## Supplementary Materials

The following supporting information can be downloaded at: https://www.mdpi.com/article/10.3390/s22228984/s1, Figures S1 to S8.
