# Supplementary material for: Benthic Fluxes of Fluorescent Dissolved Organic Material, Salt, and Heat Measured by Multiple-Sensor Aquatic Eddy Covariance
Source: Sensors (Basel). 2022 Nov 20;22(22):8984. doi: 10.3390/s22228984 (PMC9696887; doi:10.3390/s22228984)
Supplement: Supplementary file 1 [file sensors-22-08984-s001.zip › sensors-1917653-supplementary.pdf]

Hu, I. H, and H. F. Hemond. 2022. Benthic fluxes of fluorescent dissolved organic material, salt, and heat measured by multiple-sensor aquatic eddy covariance

Supplemental information

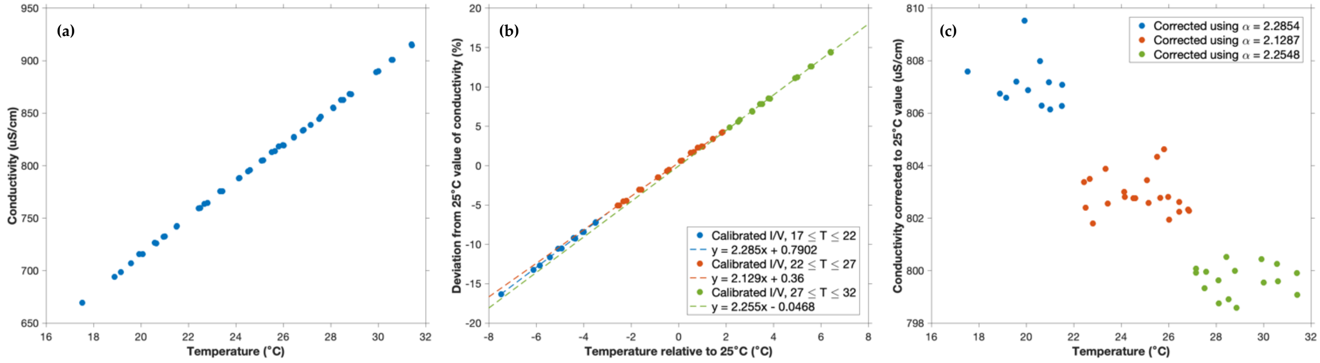

Figure S1. (a) Conductivity-temperature measurements of an incrementally heated solution of tap water and ice; **(b)** calculation of temperature compensation coefficients; and **(c)** evaluation of compensation on same data. Using fit with intercepts creates an offset when the correction is made that is removed with the mean in AEC processing, and avoids a temperature-dependent bias in the correction. Data from UML were corrected using α=2.29, and Bedford and Sunderland were corrected using α=2.13.

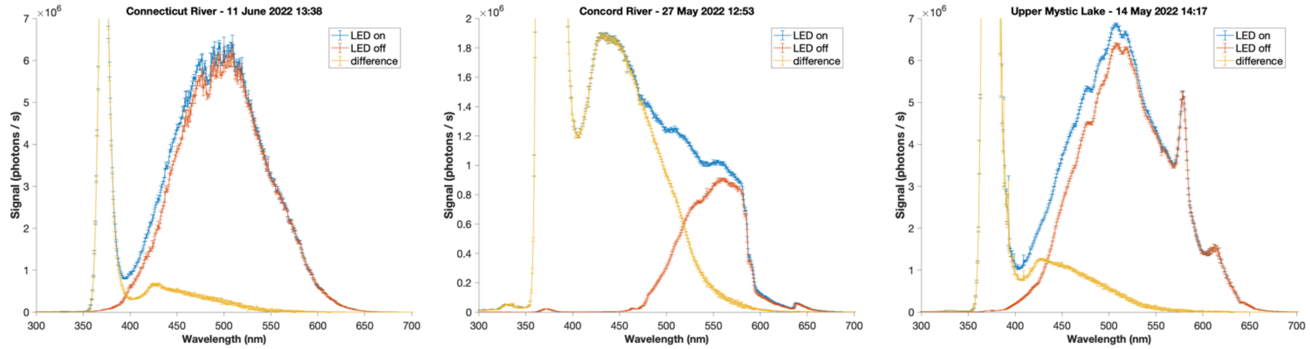

Figure S2. Spectral scans from all sites taken prior to eddy covariance deployments, showing measurements with LED on, LED off (ambient light), and the modulated difference (fluorescence) signal. Graphs show average and standard deviation over 20 trials of 20 ms each (10ms LED on, 10 ms LED off) in steps of 1 nm.

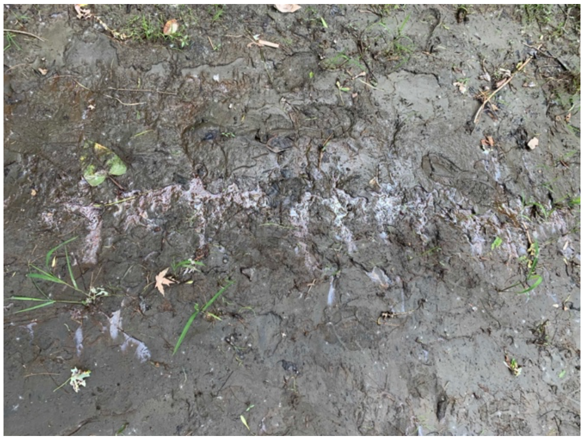

Figure S3. Precipitation of iron in exposed sediment along the bank at the Connecticut River site, illustrating both existence and spatial variability of groundwater seepage in this reach of the river.

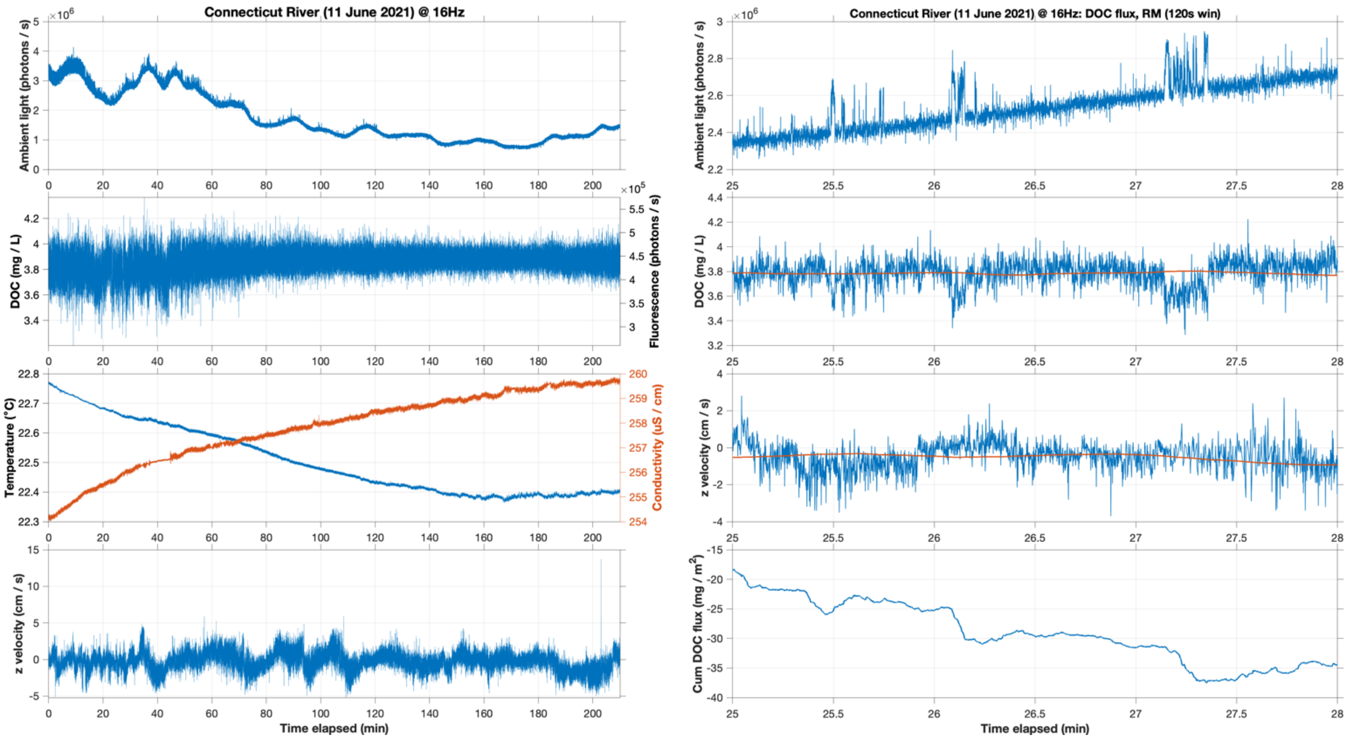

Figure S4. (left) Ambient light, FACT concentrations, and vertical water velocity measured at Connecticut River over 3.5 hour deployment. (right) Zoom of ambient light, DOC, vertical velocity, and cumulative flux, showing running means in red for DOC and velocity. Decrease in DOC signal during bursts in ambient light are apparent.

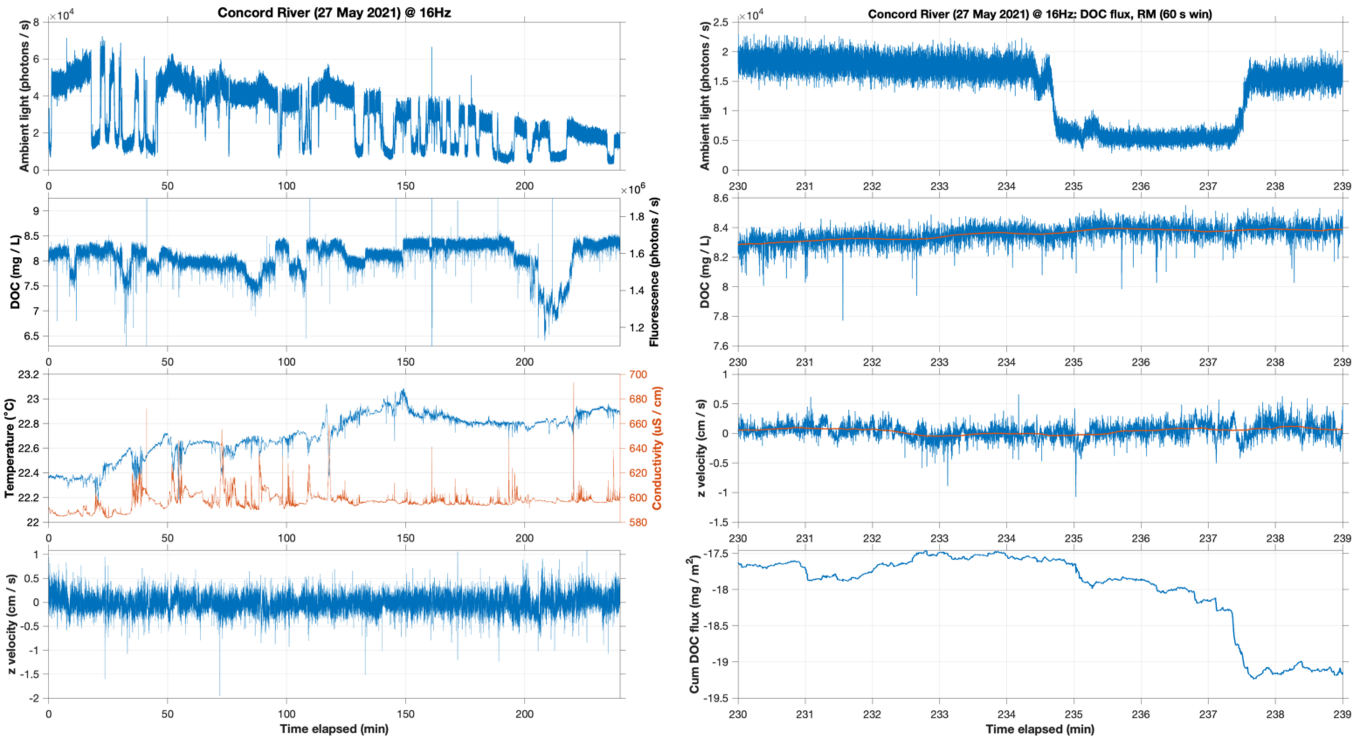

Figure S5. (left) Ambient light, FACT concentrations, and vertical velocity measured at Concord River over 4 hour deployment. (right) Zoom of ambient light, DOC, velocity, and cumulative flux, showing running means in red for DOC and velocity.

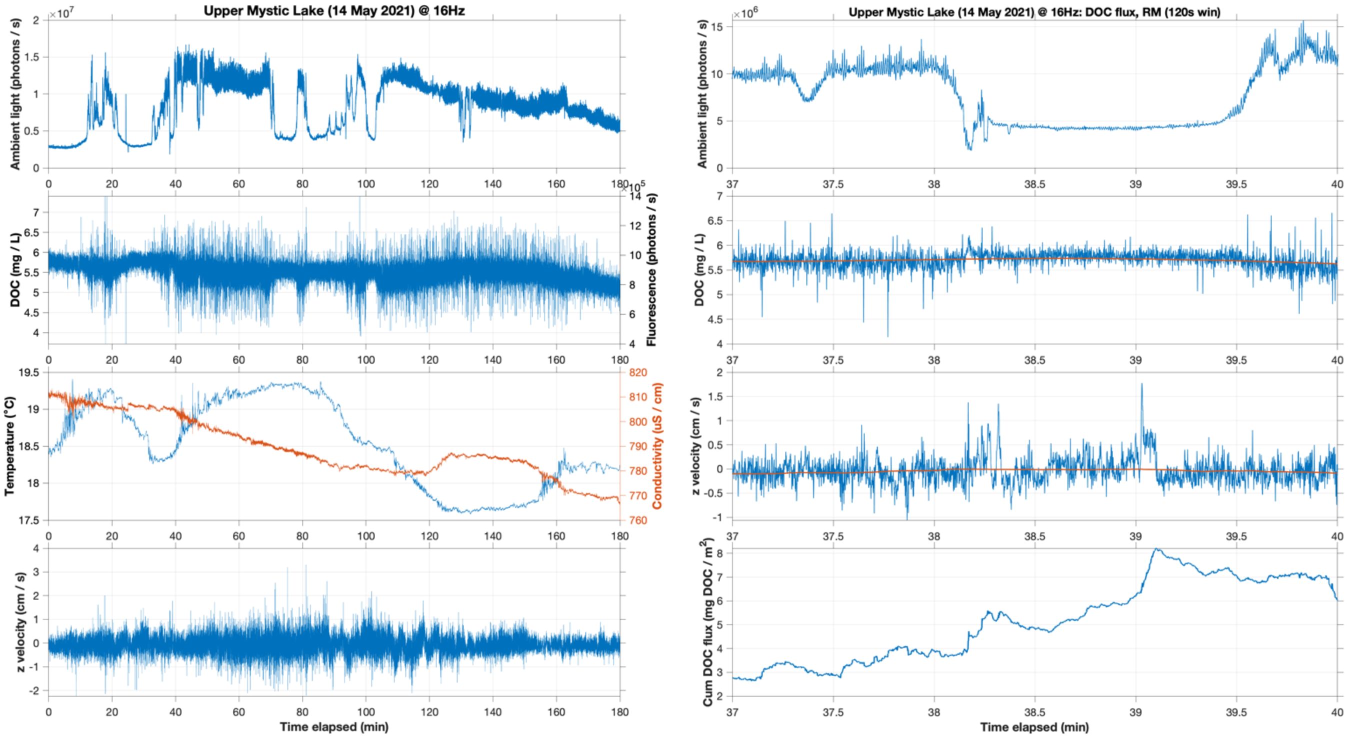

Figure S6. (left) Ambient light, FACT concentrations, and vertical velocity measured at Upper Mystic Lake over 3 hour deployment. (right) Zoom of ambient light, DOC, velocity, and cumulative flux, showing running means in red for DOC and velocity. Wave signal seen in ambient light is not apparent in DOC (fluorescence) signal.

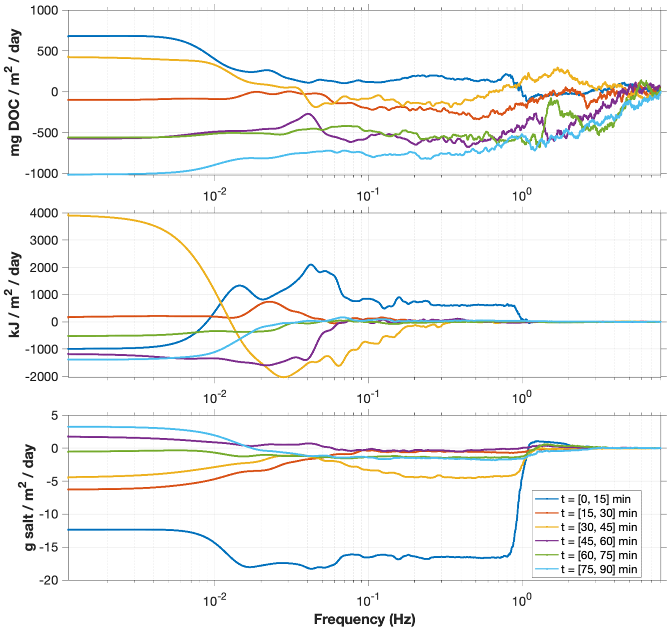

Figure S7. Cumulative cospectra of DOC flux (top), heat flux (middle), and salt flux (bottom) at Upper Mystic Lake, shown for 15 minute flux windows. Influence of wind-driven surface waves is suggested for first window by maximum in cospectra amplitude between 1 and 2 Hz, and is especially prominent for conductivity.

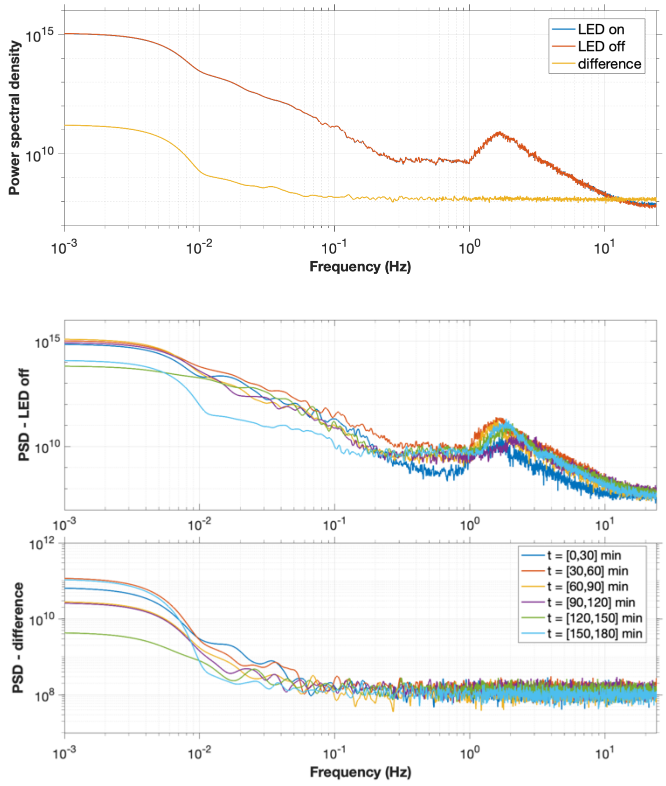

Figure S8. Power spectra for fluorescence measurements at Upper Mystic Lake. (top) Spectra of LED on, LED off, and difference signals, showing wave signal in ambient light removed by modulation. Spectra in 30 minute time periods for ambient light (middle) and difference between LED on and LED off signals (bottom) show that wave signal was removed in all time periods, regardless of strength or frequency.
